# *Vibrio alginolyticus* Triggers Inflammatory Response in Mouse Peritoneal Macrophages *via* Activation of NLRP3 Inflammasome

**DOI:** 10.3389/fcimb.2021.769777

**Published:** 2021-11-15

**Authors:** Jinxin Wang, Qun Ding, Qiankun Yang, Hui Fan, Guili Yu, Feixue Liu, Babatunde Kazeem Bello, Xiao Zhang, Tianmeng Zhang, Jingquan Dong, Gang Liu, Panpan Zhao

**Affiliations:** ^1^ Jiangsu Institute of Marine Resources Develepment, Jiangsu Key Laboratory of Marine Pharmaceutical Compound Screening, Jiangsu Key Laboratory of Marine Bioresources and Environment, Lianyungang, China; ^2^ Department of Endocrinology, The Second People’s Hospital of Lianyungang City, Lianyungang, China; ^3^ State Key Laboratory of Rice Biology, Lianyungang Academy of Agricultural Sciences, Lianyungang, China

**Keywords:** *Vibrio alginolyticus*, macrophages, NLRP3 inflammasome, IL-1β, caspase-1, inflammatory response

## Abstract

*Vibrio alginolyticus* is a food-borne marine *Vibrio* that causes gastroenteritis, otitis media, otitis externa, and septicemia in humans. The pathogenic mechanisms of *V. alginolyticus* have previously been studied in aquaculture animals; however, the underlying mechanisms in mammals remain unknown. In this study, an *in vitro* model of mouse peritoneal macrophages infected with *V. alginolyticus* was established. qPCR results revealed that *V. alginolyticus* induced the transcription levels of various cytokines, including IL-1β, IL-12, IL-18, TNF-α, IL-17, IL-6, IFN-γ, and IL-10, and the secretion level of IL-1β is the most significant. Inhibition assays with Ac-YVAD-CHO (a caspase-1 inhibitor) and Z-VAD-FMK (a pan-caspase inhibitor) were conducted to determine whether caspase-1 or caspase*-*11 is involved in *V. alginolyticus-*triggered IL-1β secretion. Results showed that IL-1β secretion was partly inhibited by Ac-YVAD-CHO and absolutely blocked by Z-VAD-FMK. To explore the sensed pattern recognition receptors, several NLR family members and the AIM2 receptor were detected and many receptors were upregulated especially NLRP3. Moreover, the NLRP3 protein displayed a puncta-like surrounding cell nucleus, which signified that the NLRP3 inflammasome was activated in response to *V. alginolyticus* infection. Inhibition assays with glyburide and CA-074 methyl ester (K^+^ outflow inhibitor and cathepsin B inhibitor) blocked IL-1β secretion, which demonstrated the essential role of the NLRP3 inflammasome in inflammatory response. To better understand how *V. alginolyticus* affects IL-1β release, the NLRP3 inflammasome was detected with doses ranging from 0.1 to 10 MOIs and time periods ranging from 3 to 12 h. Results showed that *V. alginolyticus*-mediated NLRP3 inflammasome activation was in a time- and dose-dependent manner and IL-1β release peaked at MOI of 1 for 12 h. Most importantly, blocking the NLRP3 inflammasome with inhibitors and the use of NLRP3^-/-^ and caspase-1/11^-/-^ mice could attenuate pro-inflammatory cytokine secretion, such as IL-1β, IL-6, IL-12, and TNF-α. Taken together, our study first found that the NLRP3 inflammasome plays vital roles in V. alginolyticus triggered inflammatory response in mouse peritoneal macrophages. This may provide reference information for the development of potential anti-inflammatory treatments against *V. alginolyticus* infection.

## Introduction

*Vibrio alginolyticus* (*V. alginolyticus*), a Gram-negative marine *Vibrio*, is widely distributed in the ocean and offshore coastal and estuarine areas. According to an increasing number of studies, *V. alginolyticus* is not only limited to infecting marine species such as oysters, groupers, and *Litopenaeus vannamei* (Liu et al., 2004; Wang et al., 2016; Mohamad et al., 2019) but is also emerging as an opportunistic pathogen infecting humans. Infection with *V. alginolyticus* is primarily associated with inflammatory disorders in humans, for example gastroenteritis, otitis media, otitis external, and septicemia (Uh et al., 2001; Feingold and Kumar, 2004; Issack et al., 2008; Gaüzère et al., 2016). Cases of *V. alginolyticus* infection have been reported around the world, including Europe, Asia, South, and North America. Ingestion of *V. alginolyticus*-contaminated raw seafood or wound exposed to *V. alginolyticus*-containing warm saltwater is both related to the bacteria infection. To solve this problem, antibiotics are widely used and the abuse of traditional antibacterial drugs leads to antibiotic resistance (Zavala-Norzagaray et al., 2015; Hernández-Robles et al., 2016; Kang et al., 2016), thereby making such infections difficult to be controlled. Therefore, an in-depth study into the pathogenic mechanism is of great significance to preventing and controlling *V. alginolyticus*-caused diseases.

The innate immune system is the host’s first defense line to fight against pathogens. Phagocytes, including macrophages and neutrophils, play necessary roles in resistance to pathogen invasion. Macrophages, as “scavengers” in the body, are mainly responsible for ingesting pathogenic bacteria and removing cell debris. This function is mainly attributed to the various cell-surface pattern recognition receptors (PRRs), which can recognize pathogen-associated molecule patterns (PAMPs) or danger-associated molecular patterns (DAMPs) released by exogenous invaders (Medzhitov, 2007; Zhang and Wang, 2014). According to previous research reports on *Vibrio*, such as *Vibrio parahaemolyticus* (*V. parahaemolyticus*), *Vibrio vulnificus* (*V. vulnificus*), and *Vibrio cholerae* (*V. cholerae*), inflammatory infection models are all established using macrophages (Higa et al., 2013; Huang X. H. et al., 2020; Mamantopoulos et al., 2019). Nonetheless, most studies on *V. alginolyticus* pathogenic cases are focused on aquaculture animals (Huang M. et al., 2020). Therefore, as an important zoonotic pathogen, the potential mechanism of mammalian macrophages’ immune response to *V. alginolyticus* infection still needs to be explored further.

Inflammation is the immediate response of the host’s innate immune cells to the damage caused by pathogens; chemical, physical, and other harmful stimuli, or bodily injuries. Inflammasomes play critical roles in inflammatory response. The main types of inflammasome include NLRP1, NLRP3, NLRC4, AIM2, and Pyrin. The type of NLRP3 as the most classical inflammasome has been extensively studied. It is a large supramolecular protein and composed of a sensor molecule (NLRP3 protein), the adaptor apoptosis­associated speck­like protein (ASC), and the effector protease caspase-1 (Sharma and Kanneganti, 2016; Yang et al., 2019). Recent research has shown that NLRP3 inflammasomes also play an important role in inflammation-related diseases such as cancer, atherosclerosis, diabetes, obesity, and vibriosis (Toma et al., 2010; Sharma and Kanneganti, 2021; Yu et al., 2021). Activation of NLRP3 inflammasomes requires two signals. The initial signal is the activation of MAPK or/and NF-κB mediated by toll-like receptors (TLRs), which produces not only the cytokines of IL-6, IL-12, and TNF-α but also the pro-IL-1β and NLRP3 proteins. The stimulants trigger the secondary signal of NLRP3 inflammasome activation, including changes in intracellular potassium concentration, production of reactive oxygen species, lysosomal disruption to release cathepsin B, or mitochondrial dysfunction. When activation signals are generated, these three domains will quickly assemble and produce caspase-1/p20, an executive protein with cleavage function, to cleave the precursor of IL-1β to produce mature IL-1β and release outside the cell, leading to inflammatory response (Latz and Duewell, 2018). Pathogenic vibrios of *V. vulnificus* and *V. cholerae* induced the production of inflammatory cytokine IL-1β by activating the NLRP3 inflammasome in mouse bone marrow-derived macrophages (Toma et al., 2010). Growing evidence shows that most *V. alginolyticus* infections are closely related to inflammatory diseases. However, it is unknown whether inflammasomes have a role in the inflammation caused by *V. alginolyticus* and how inflammasomes function. Therefore, an in-depth understanding on how *V. alginolyticus* activates inflammasomes would be of great benefit to reveal the inflammatory responses mechanism and provide therapeutic strategies for treating the infections.

In order to explore the mechanisms of *V. alginolyticus*-mediated inflammation, an *in vitro* model of mouse peritoneal macrophages infected by *V. alginolyticus* was established in this study. Various inflammatory cytokines were detected in *V. alginolyticus-*infected macrophages. The roles of caspase-1 p20 in IL-1β release were determined. Upregulation of PRRs was screened, and the vital NLRP3 receptor was characterized. To further understand the involvement of the NLRP3 inflammasome in *V. alginolyticus*-induced inflammation, specific inhibitors, NLRP3^-/-^ and caspase-1/11^-/-^, in mice were used to block the activation of NLRP3 inflammasome and determined its roles in the secretion of other inflammatory cytokines.

## Materials and Methods

### Materials and Reagents

*V. alginolytic*us ATCC 17749 was purchased from American Type Culture Collection (ATCC, Manassas, VA, USA). Caspase-1 (p20, AG-20B-0042) and NLRP3 (AG-20B-0014) were purchased from AdipoGen Life Sciences (Liestal, Switzerland). IL-1β (p17) was purchased from R&D (AF-401-SP, Minnesota, USA). Ac-YVAD-CHO was purchased from Enzo Life Sciences (ALX-260-027, Lausen, Switzerland). Z-VAD-FMK and CA-074 methyl ester were obtained from Selleck (S7023, S7042, Shanghai, China). Glyburide was obtained from MedChemExpress (HY-15206, St. New Jersey, USA). RIPA was purchased from Solarbio (R0020, Beijing, China). RPMI 1640 (01-100-1ACS), fetal bovine serum (FBS, 04-001-1ACS), and gentamicin (03-035-1C) were all obtained from Biological Industries (Israel). The TRIzol reagent was purchased from Monad (MI20101S, Wuhan, China). The LDH Cytotoxicity Assay Kit was obtained from Beyotime (C0016, Shanghai, China).

### Animals and Preparation of Peritoneal Macrophages

All the animal experiments were conducted in accordance with the requirements of the Animal Welfare and Research Ethics Committee, Jiangsu Ocean University, certificate number 2019220341. Six- to 8-week-old female C57BL/6 mice were purchased from Pizhou Oriental Breeding Company (Pizhou, China). NLRP3*^-/-^* mice were purchased from The Jackson Laboratory (ME, USA), and Caspase-1/11^-/-^ mice were provided by Dr. Feng Shao (Beijing, China). Each mouse was intraperitoneally injected with 2.5 ml of the 2.98% Difco Fluid Thioglycolate Medium (BD, USA) 3–4 days in advance. Then, they were humanely euthanized by cervical dislocation and soaked in 75% alcohol for 5 min. Phosphate-buffered solution (PBS) was used to lavage mice’s abdominal cavity to isolate macrophages. Cells were washed three times with PBS and centrifuged at 1,000 × g for 10 min. The cell precipitation was resuspended in 1 ml of RPMI 1640 medium supplemented with 10% FBS and 1% penicillin–streptomycin. Ten microliters of cell suspension was diluted for cell counting using the counting formula: cell count/mL=cell count/80 × 400 × 10^4^ × dilution multiple. According to the counting results, 4.5 × 10^6^ cells/well were added into six-well plates and cultured in an incubator containing 5% CO_2_ at 37°C for 4–6 h. After sedimentation, unattached cells were discarded and the culture medium was replaced with RPMI 1640 medium supplemented with 1% FBS and 1% penicillin–streptomycin, and cells were continued to culture for 12 h and then used for subsequent experiments. The cell purity was verified with APC anti-mouse/human CD11b (1:200, BioLegend, USA) *via* flow cytometry.

### Establishment of Mouse Macrophages Infection Model by *V. alginolyticus*

*V. alginolyticus* was enriched in alkaline peptone water (APW) under conditions of 30°C/200 rpm. *V. alginolyticus* at a logarithmic growth period (OD_600 nm_ = 0.6) was counted, washed three times in sterile PBS, and then resuspended in RPMI 1640 medium supplemented with 1% FBS. Peritoneal macrophages were infected with *V. alginolyticus* at different multiples of infection (MOI). After incubating for 3 h, *V. alginolyticus* were removed followed by washing three times with PBS. Then, the cells were incubated in RPMI 1640 medium supplemented with 1% FBS and 3 µg/ml gentamicin at 37°C for 3, 6, or 12 h.

### Quantitative Real-Time PCR

Total RNA was extracted from cell pellets using the TRIzol reagent according to the manufacturer’s recommendations. One microliter of total RNA was diluted 10 times using diethyl pyrocarbonate (DEPC) water, and the concentration and purity were measured with the NanoDrop ND-2000 apparatus (Thermo Scientific, USA). Two to three micrograms of total RNA was reverse transcribed to cDNA and then amplified using MonAmp SYBR Green qPCR Mix (None ROX, China). The quantitative real-time PCR (qPCR) protocol was set as follows: the initial cDNA template was denatured at 95°C for 30 s, followed by amplification of the cDNA template for 40 cycles, in which the amplified cycles include denaturation at 95°C for 10 s, primer annealing, and extension at 60°C for 30 s. qPCR assays were performed on a LightCycler 480 II machine (Roche, Germany). Relative mRNA fold changes were normalized to an internal control of β-actin. The data were calculated by the comparative Ct pattern (2^-ΔΔCt^ formula). Primers were synthesized from Sangon Biotech (Shanghai, China), and the primer sequences are listed in **Table 1**.

**Table 1:** qPCR primer sequences of inflammatory cytokines and NLRs.

Name	Accession number	Primer sequence (5′ to 3′)
IL-6	NC_000071	F: TGCCTTCTTGGGACTGATGC
		R: GCAAGTGCATCATCGTTGTTC
IL-10	NC_000067	F: GCAGTGGAGCAGGTGAAGAG
		R: CGGAGAGAGGTACAAACGAGG
IL-12	MMU23922	F: TACAAGGTTCAGGTGCGAGC
		R: ATGTATCCGAGACTGCCCAC
IL-18	NM_008360	F: ACCAAGTTCTCTTCGTTGAC
		R: CTTCACAGAGAGGGTCACAG
IL-1β	NM_008361	F: AGGAGAACCAAGCAACGACA
		R: CTCTGCTTGTGAGGTGCTGA
TNF-α	NM_013693	F: GACGTGGAACTGGCAGAAGA
		R: GGCTACAGGCTTGTCACTCG
IFN-γ	NM_008337	F: CGGCACAGTCATTGAAAGCC
		R: TGTTGTTGCTGATGGCCTGA
NOD1	NM_172729.3	F: GATTGGAGACGAAGGGGCAA
		R: CGTCTGGTTCACTCTCAGCA
NOD2	NM_145857.2	F: GCCAGTACGAGTGTGAGGAG
		R: GCGAGACTGAGTCAACACCA
NLRP1	NC_000077	F: ATAAACAAGCCACCCCCAGT
		R: TGTGCCCAATGTCGATCTCA
NLRP2	NC_000073	F: AGGCGGTCTTTCCAGAGAATG
		R: TCCAGTGCAGAGCTGTTGAG
NLRP3	NM_145827.4	F: AGCCAGAGTGGAATGACACG
		R: CGTGTAGCGACTGTTGAGGT
NLRP6	NM_133946.2	F: TTGTTCGACAGGCTCTCAGC
		R: ACTGGGGGTTGTTTCTTGGT
NLRC4	NM_001033367	F: GCTCAGTCCTCAGAACCTGC
		R: ACCCAAGCTGTCAATCAGACC
NLRC5	NM_001033207	F: TCTCTAAGCAGCTAGGGGCA
		R: GGGGAGTGAGGAGTAAGCCA
β-actin	NM_007393	F: GCCATGTACGTAGCCATCCA
		R: ACGCACGATTTCCCTCTCAG

F, forward primer; R, reverse primer.

### Western Blotting

The culture supernatants were collected and concentrated as previously described (Zhao et al., 2021). Cell pellets were washed in ice-cold PBS and lysed with RIPA containing the protease inhibitor of phenylmethanesulfonyl fluoride (PMSF, 1 mM). The protein samples were quantified with a BCA Protein Assay Kit (Thermo Scientific, USA) according to the manufacturer’s protocol. Each sample (30 μg) of cell lysates or cell supernatants (10 μl) were separated by 12% SDS-PAGE, and the electrophoresis protocol was set as follows: 80 V 1 h 05 min and 120 V 30 min. Then, the protein was transferred onto 0.22 or 0.45 μm PVDF membrane (Millipore, USA) under conditions of 200 mA/1 h or 1 h 30 min at 4°C. The membranes were blocked in 5% bovine serum albumin (BSA) in PBST for 2 h at room temperature and incubated at 4°C overnight with different primary antibodies (Abs), including IL-1β (1:1,000), caspase-1 (1:1,000), NLRP3 (1:1,000), and β-actin (1:5,000). The HRP-conjugated secondary antibodies were rabbit anti-goat IgG (H+L) (1:5,000) or goat anti-mouse IgG (H+L) (1:5,000, EarthOx, USA). The blots were developed using the ChemiScope Western Blot Imaging System (Clinx Science Instruments, China).

### Enzyme-Linked Immunosorbent Assay

The culturing supernatants were collected from infected cells, centrifuged to remove cell precipitation, and stored at -80°C for subsequent experiments. ELISA Kits (Invitrogen, USA) were used to analyze the secretion levels of IL-1β, IL-6, IL-12, and TNF-α in the supernatants according to the instructions provided by the manufacturer. The absorbance at 450 nm was read using a microplate reader (BioTek, USA). The OD values were converted to pg/mL according to the standard curve.

### LDH Level Measurement

To explore whether *V. alginolyticus* triggered cell death in mouse macrophages, the LDH levels were tested in infected cell culture supernatant at 12 h (MOI = 0.1, 0.5, 1, or 10). The absorbances were read under 490- and 600-nm wavelength according to the manufacturer’s recommendations.

### Immunofluorescence Staining

Macrophages previously prepared on coverslips in 24-well plates were inoculated with *V. alginolyticus* (MOI = 1) for 3 h, and then the culture medium was replaced by RPMI 1640 medium supplemented with 1% FBS and 3 µg/ml gentamicin after washing three times with PBS. After 12 h, the infected cells were fixed in 4% paraformaldehyde (Biosharp, Beijing), and then the cell membrane was penetrated in 0.5% Triton X-100. Cells were blocked in 5% BSA at room temperature for 2 h and incubated with NLRP3 Rabbit pAb (1:100) at 4°C overnight. Then, cells were incubated with an FITC-labeled secondary antibody of goat anti-mouse (H+L) (1:200) at 37°C for 1 h; the nucleus was then stained with 1 μg/ml 4′,6-diamidino-2-phenylindole, dihydrochloride (62247, DAPI, Thermo Scientific, USA) as recommended by the manufacturer. The coverslips were fixed to Adhesion Microscope Slides (Servicebio, China), and the fluorescence images were observed on the fluorescence microscope (Olympus, Japan).

### Inhibitor Experiment

Inhibitors of Ac-YVAD-CHO, Z-VAD-FMK, CA-074 methyl ester, and glyburide were prepared in the appropriate storage solutions according to the product instructions and stored at -80°C. The inhibitors were cocultured with peritoneal macrophages for 1 h prior to stimulation. The corresponding final concentrations are as follows: Ac-YVAD-CHO (100 μM), Z-VAD-FMK (10 μM), CA-074 methyl ester (25 μM), and glyburide (50 μM). Then, the inhibitor was discarded and washed three times with sterile PBS and replaced with fresh medium. Subsequently, the cells were infected with *V. alginolyticus* for 3 h and the culture medium was replaced by RPMI 1640 medium supplemented with 1% FBS and 3 µg/ml gentamicin after washing three times with PBS. After 12 h, cells were collected for qPCR analysis and Western blotting. The supernatants were used for ELISA assays.

### Statistical Analysis

The results of the bar chart were presented through GraphPad Prism 8 (Inc., La Jolla, USA) with mean ± standard deviation (SD). The gray value comparison results were analyzed by ImageJ (National Institutes of Health, USA). The dataset comparisons were performed with IBM SPSS Statistics 20 (Chicago, IL), and there were two cases: the independent sample *t*-test was used to compare between two groups of data; the data of three groups and above were analyzed by one-way ANOVA, and the uniformity of variances was taken as Bonferroni or the inhomogeneity of variances was taken as Tamhane’s T2. All experiments were conducted at least three times with representative results shown in the figures. Data were regarded as statistical significance when ^*^ *p* < 0.05, ^**^ *p* < 0.01, ^***^ *p* < 0.001, and not significant (ns), *p* > 0.05.

## Results

### *V. alginolyticus* Induces Inflammatory Response in Macrophages

**Figure 1:**
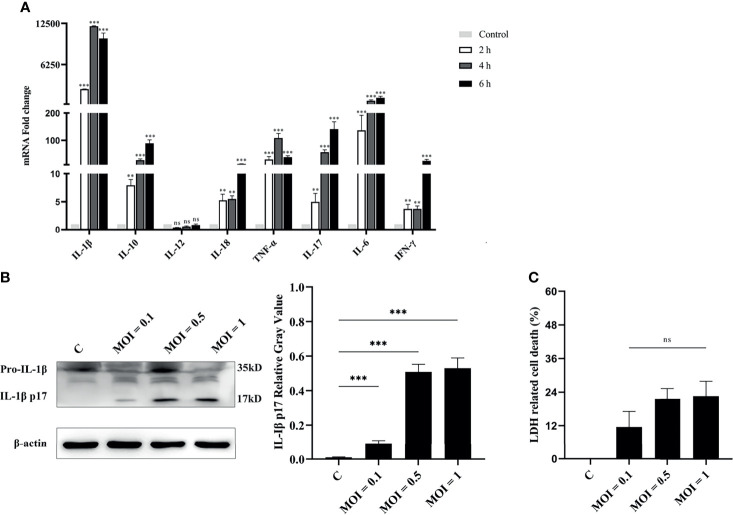
*V. alginolyticus* induced pro-inflammatory cytokine secretion in mouse peritoneal macrophages. **(A)** Macrophages were infected with *V. alginolyticus* at an MOI of 1 for 2, 4, and 6 h Then, the mRNA levels of various inflammatory cytokines such as IL-1β, IL-6, IL-12, TNF-α, IL-17, IL-18, and IFN-γ and anti-inflammatory cytokine IL-10 were tested using qPCR. **(B)** Macrophages were infected with *V. alginolyticus* at MOIs of 0.1, 0.5, and 1 for 12 h, and the protein expression levels of IL-1β p17 in the cell supernatants were examined by Western blotting. The relative gray value of IL-1β p17 was normalized to β-actin in the cell lysates. **(C)** Cell death was monitored by measuring LDH release in supernatants after infection with *V. alginolyticus* for 12 h. Data were representative of at least three independent experiments. ns, not significance, **p < 0.01, and ***p < 0.001.

Exposure of RAW264.7 macrophages to *V. parahaemolyticus* triggered inflammatory response by secretion of inflammation-related cytokines, such as IL-1β, IL-6, and TNF-α (Gulati et al., 2019). Therefore, the secretion of inflammatory cytokines in *V. alginolyticus*-infected peritoneal macrophages was first measured. The qPCR data showed that the transcriptional levels of various inflammatory cytokines in the lysates of macrophages were obviously upregulated (^**^ *p* < 0.01 or ^***^ *p* < 0.001) at 2, 4, and 6 h except for IL-12, and IL-1β occupied the highest expression level (**Figure 1A**). To further determine the secretion of IL-1β, the protein expression levels of IL-1β/p17 were detected in cell supernatants collected from infected cells at MOI = 0.1, 0.5, and 1. Western blotting combined relative gray values to β-actin data, suggesting that the IL-1β p17 protein expression levels in different infection doses were significantly higher than those in the untreated control (C) group (^***^ *p* < 0.001). Moreover, the relative gray value data showed that the protein levels of IL-1β p17 continued to increase with the increase in infectious dose (**Figure 1B**). In addition, the release of LDH, a marker of lytic cell death, occurred in response to *V. alginolyticus* stimulation with no obvious change under different MOI treatments (**Figure 1C**). These data together indicated that *V. alginolyticus* infection triggered inflammation and induced the release of IL-1β.

### Activation of Caspase-1 Is Essential for IL-1β Secretion

**Figure 2:**
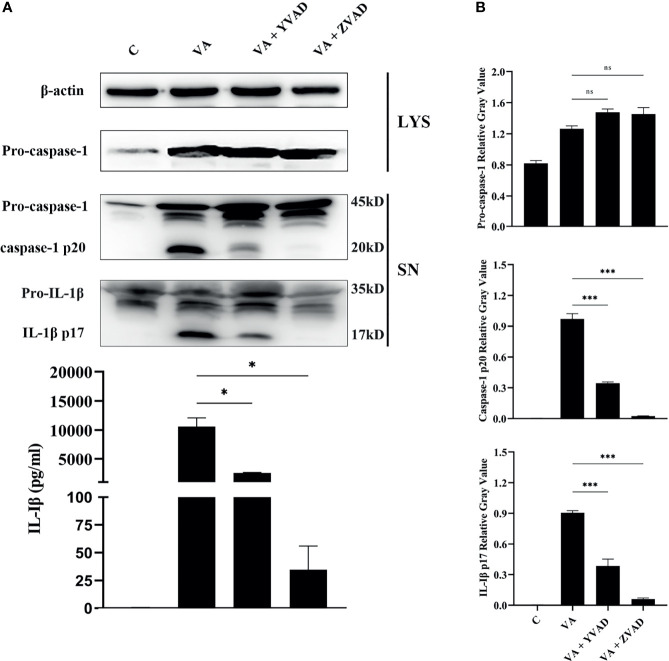
IL-1β release was dependent on the activation of caspase-1 after *V. alginolyticus* infection. **(A, B)** Macrophages were pretreated with 100 μM Ac-YVAD-CHO or 10 μM Z-VAD-FMK for 1 h and then infected with *V. alginolyticus* with MOI of 1 for 12 h; the protein expression levels of pro-caspase-1, caspase-1 p20, and IL-1β p17 were detected by Western blotting, and the IL-1β level band gray value with ImageJ, and the relative gray value was calculated through normalizing to β-actin. Data from ELISA and Western blotting results were representative of at least three independent experiments. *p < 0.05, ***p < 0.001, ns, not significance.

Previous studies confirmed that *V. parahaemolyticus* infection induced IL-1β release by activation of caspase-1 in mouse bone marrow-derived macrophages (Higa et al., 2013). To test whether IL-1β secretion depends on the classical caspase-1 cleavage pathway or the non-classical caspase-11 cleavage pathway, the secretion of IL-1β or activation of caspase-1 in the culturing supernatants and the expression of pro-caspase-1 in cell lysates were detected by Western blotting. Results showed that after treatment with the caspase-1 inhibitor of Ac-YVAD-CHO or pan-caspase inhibitor of Z-VAD-FMK, caspase-1/p20 activation was significantly inhibited (^***^ *p* < 0.001). Interestingly, no obvious changes were found in the expression of pro-caspase-1 in cells (*p* > 0.05). Inhibition of caspase-1 activation significantly led to decrease in IL-1β/p17 secretion (^***^ *p* < 0.001) in comparison to the *V. alginolyticus* infection group. This was consistent with the mRNA expression levels of IL-1β measured by qPCR (^***^ *p* < 0.001) and IL-1β secretion levels detected by ELISA (^*^ *p* < 0.05, **Figure 2** and **Figure S1**). Overall, these data manifested that the IL-1β release was dependent on the activation of caspase-1 in *V. alginolyticus* infected mice macrophages.

### NLRP3 Inflammasome Assembly in Response to *V. alginolyticus* Infection

**Figure 3:**
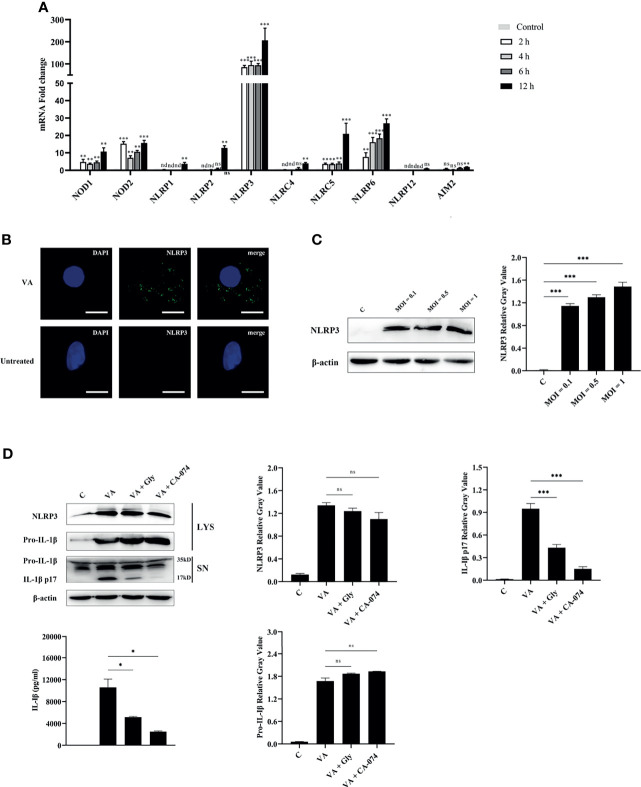
NLRP3 inflammasome was activated in *V. alginolyticus*-infected macrophages and played vital roles in IL-1β secretion. **(A)** Macrophages were infected with *V. alginolyticus* at an MOI of 1 for 2, 4, 6, and 12 h, and then the mRNA levels of patterns recognize receptors including NOD1, NOD2, NLRP1, NLRP2, NLRP3, NLRC4, NLRC5, NLRC6, NLRP12, and AIM2 were detected using qPCR. Data represented the mRNA fold change compared with the control group standardized to β-actin. **(B)** Mouse peritoneal macrophages were infected with *V. alginolyticus* (MOI = 1) for 12 h, and then the localization of NLRP3 protein was visualized by immunofluorescent staining. The green signals represent FITC-labeled NLRP3 protein, and the blue signals represent DAPI-stained nucleus. **(C)** Macrophages were infected for 12 h with *V. alginolyticus* at MOIs of 0.1, 0.5, and 1. The levels of NLRP3 in the cell lysates were detected by Western blotting, and the relative gray value was analyzed using ImageJ. **(D)** Mouse peritoneal macrophages were pretreated with 50 μM glyburide or 25 μM CA-074 methyl ester for 1 h and then infected with *V. alginolyticus* at a MOI of 1 for 12 h The protein expression levels of NLRP3, pro-IL-1β, and IL-1β were examined by Western blotting combined relative gray analysis, and the IL-1β levels in the cell supernatants were also detected by ELISA. Data were representative of at least three repetitive experiments. *p < 0.05, **p < 0.01, ***p < 0.001, ns, not significance, and nd, not detected.

PRRs were the key molecules in recognizing PAMPs or DAMPs; the transcription levels of central molecules of different inflammasome families were detected in macrophages at 2, 4, 6, and 12 h after *V. alginolyticus* infection. The qPCR data showed that the expression levels of NOD1, NOD2, NLRP3, NLRC5, and NLRP6 in peritoneal macrophages significantly elevated at 2, 4, 6, and 12 h (^**^ *p* < 0.01 or ^***^ *p* < 0.001). The gene levels of NLRP1, NLRP2, NLRC4, and AIM2 markedly increased at 12 h (^**^ *p* < 0.01), and the transcription levels of NLRP12 remained unchanged at 12 h (*p* > 0.05). On the basis of these data, the NLRP3 receptor upregulated to the highest levels when compared with other NLR family members including the AIM2 receptor (**Figure 3A**). To further explore whether the NLRP3 inflammasome was assembled during *V. alginolyticus* infection, the subcellular localization of the NLRP3 protein was observed by immunofluorescence staining. Images captured by fluorescence microscopy showed that NLRP3 aggregated in the perinuclear region after infection with *V. alginolyticus*, whereas no signals were observed in untreated cells (**Figure 3B**). In order to confirm the NLRP3 protein expression levels, Western blotting assays were conducted to detect the expression of NLRP3 protein at different infection doses (MOI = 0.1, 0.5, and 1). Relative gray values showed that the NLRP3 protein was significantly upregulated at various infection doses compared to the control group (***p < 0.001) and no protein was detected in the C group (**Figure 3C**). To further explore the activating ways of NLRP3 inflammasome and its roles in the regulation of IL-1β during *V. alginolyticus* infection, cells were pretreated with glyburide or CA-074 methyl ester prior to stimulation and the protein expression levels of NLRP3 and IL-1β were detected using Western blotting and ELISA assays. The protein expression levels of IL-1β p17 significantly decreased in both inhibition groups when compared to the *V. alginolyticus* infection group (^***^ *p* < 0.001); in contrast, the protein expression levels of pro-IL-1β and NLRP3 in cells displayed no significant differences between the experiment group and the inhibitor-treated groups (*p >* 0.05). ELISA results also indicated that both glyburide and CA-074 methyl ester downregulated the IL-1β protein secretion levels (^*^ *p* < 0.05) and CA-074 methyl ester reduced IL-1β to relatively lower levels, suggesting that *V. alginolyticus* infection mainly activates the NLRP3 inflammasome through the release of cathepsin B (**Figure 3D**).

Similarly, the results of qPCR are indistinguishable from those of western blotting and ELISA (^***^ *p* < 0.001, **Figure S2**). Taken together, these data revealed that *V. alginolyticus* induced the release of the proinflammatory cytokine of IL-1β in mouse peritoneal macrophage, which was highly dependent on the activation of NLRP3 inflammasome.

### Activation of NLRP3 Inflammasome Is in a Dose-Dependent and Time-Dependent Manner

**Figure 4:**
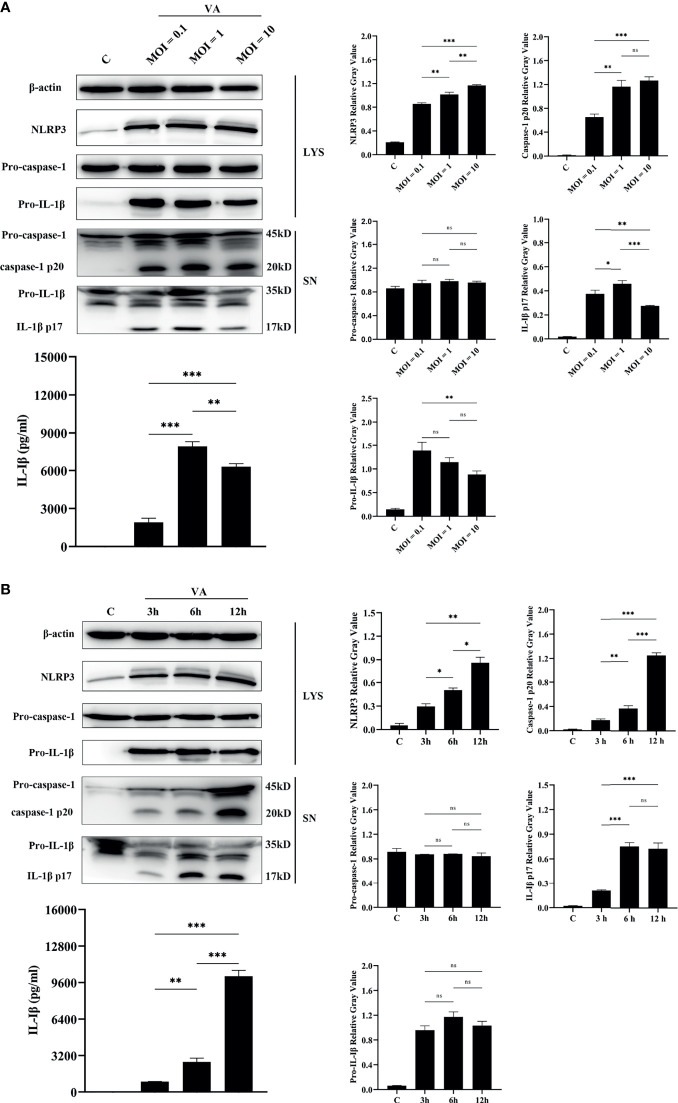
The activation of the NLRP3 inflammasome was dose-dependent and time-dependent. **(A)** Dose-dependent (MOI = 0.1, 1, and 10) assay of macrophages infected with *V. alginolyticus* for 12 h The protein expression levels of NLRP3, pro-caspase-1, caspase-1, pro-IL-1β, and IL-1β were confirmed by Western blotting, and IL-1β protein expression was also detected by ELISA. **(B)** Time course (3, 6, and 12 h) assay of macrophages infected with *V. alginolyticus* at MOI of 1. The protein expression levels of NLRP3, pro-caspase-1, caspase-1, pro-IL-1β, and IL-1β were confirmed by Western blotting, and the mature IL-1β released into supernatant was detected by ELISA. Data from ELISA and Western blotting gray value results were representative of at least three repetitive experiments. ns, not significance, *p < 0.05, **p < 0.01, and ***p < 0.001.

To better make sense of the relationship between activation of NLRP3 inflammasome and infection by *V. alginolyticus*, an experiment with *V. alginolyticus* on different infection doses was conducted to detect the secretion of IL-1β in peritoneal macrophages using qPCR, Western blotting, and ELISA methods. The grayscale analysis results showed that the protein expression levels of NLRP3 were significantly upregulated in a dose-dependent manner along with the increased infection dose of *V. alginolyticus* (^**^ *p* < 0.01 or ^***^ *p* < 0.001). The qPCR data showed that *V. alginolyticus* could induce the upregulation of IL-1β mRNA transcription at different infection doses (^***^ *p* < 0.001) and the levels showed a trend of increasing and then decreasing (**Figure S3A**). Similar results were found in the protein expression levels of IL-1β p17 in the supernatants (^*^ *p* < 0.05, ^**^ *p* < 0.01, or ^***^ *p* < 0.001), although pro-IL-1β levels remained unchanged at MOI = 1 *vs.* MOI = 0.1 and MOI = 1 *vs.* MOI = 10 (*p >* 0.05). In addition, caspase-1 p20 in the infected supernatants was significantly upregulated at MOIs of 1 and 10 compared to MOI of 0.1 (^**^ *p* < 0.01 or ^***^ *p* < 0.001), and the levels remained the same when MOIs of 1 and 10 were used; on the contrary, there were no noticeable changes in pro-caspase-1 levels in the cell precipitation (*p >* 0.05, **Figure 4A**).

The infection dose of MOI = 1, which secreted the highest levels of IL-1β p17, was selected for the different inoculation time assays. Western blotting combined with relative gray value analysis data showed that the protein expression levels of NLRP3 were significantly upregulated in a time-dependent manner during the monitored infection time of *V. alginolyticus* (^*^ *p* < 0.05 or ^**^ *p* < 0.01). Meanwhile, the secretion of mature IL-1β was in a time-dependent manner, with the maximum amount at 12 h, as indicated by Western blotting and ELISA (^**^ *p* < 0.01 or ^***^ *p* < 0.001). However, the protein levels of pro-IL-1β in the cell lysates did not vary significantly (*p >* 0.05) and qPCR data showed that IL-1β mRNA expression markedly spiked at 3 h during exposure to *V. alginolyticus* and then remained in a continuous downward trend at 6 and 12 h (^***^ *p* < 0.001, **Figure S3B**). The activation of caspase-1 p20 was positively correlated with the secretion amounts of IL-1β p17, which significantly increased during the monitored time period of 3 to 12 h, and there are significant differences at different times (^**^ *p* < 0.01 or ^***^ *p* < 0.001), but pro-caspase-1 expression levels in cells remained steady (*p >* 0.05, **Figure 4B**). Generally, these results confirm that the activation of the NLRP3 inflammasome was accumulated when infection time and dose were increased within a certain range.

### Blocking the NLRP3 Inflammasome Downregulated *V. alginolyticus*-Triggered Inflammatory Response in Mouse Macrophages

**Figure 5:**
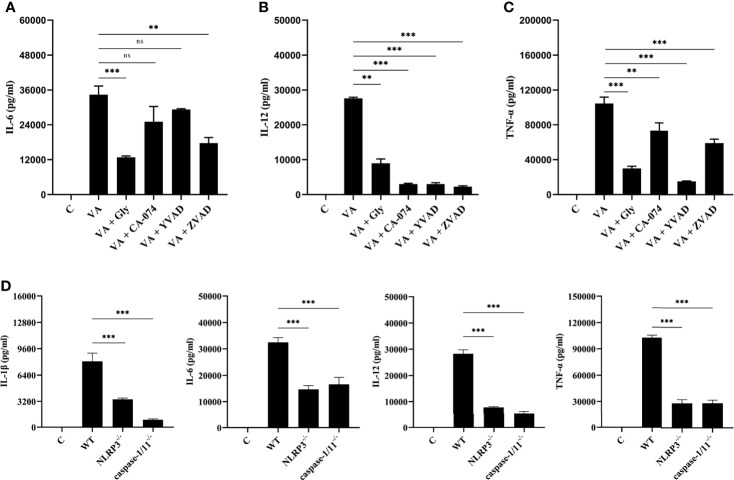
Activation of NLRP3 inflammasome positively regulated *V. alginolyticus*-mediated inflammatory response. **(A–C)** Mouse peritoneal macrophages were pretreated with 50 μM glyburide, 25 μM CA-074 methyl ester, 100 μM Ac-YVAD-CHO, or 10 μM Z-VAD-FMK for 1 h prior to *V. alginolyticus* infection under conditions of MOI = 1/12 h. The protein expressions of IL-6, IL-12 and TNF-α were measured by ELISA. **(D)** The WT, NLRP3^-/-^, and caspase-1/11^-/-^ mouse peritoneal macrophages were simulated by *V. alginolyticus* under conditions of MOI = 1/12 h. The protein expressions of IL-1β, IL-6, IL-12, and TNF-α were measured by ELISA. Data from ELISA assays were representative of at least three repetitive experiments. ns, not significance, **p < 0.01 and ***p < 0.001.

*V. alginolyticus*-mediated macrophages inflammation upregulated the expression of various pro-inflammatory cytokines. Therefore, inhibition combined with ELISA assays was performed to detect the protein release levels of IL-6, IL-12, and TNF-α. It appeared that the IL-6, IL-12, and TNF-α protein release levels were significantly decreased in inhibitor groups (^**^ *p* < 0.01 or ^***^ *p* < 0.001) except for IL-6 levels in CA-074 methyl ester and Ac-YVAD-CHO pretreatment groups (*p >* 0.05, **Figures 5A–C**). To further determine the roles of the NLRP3 inflammasome, the protein production levels of IL-1β, IL-6, IL-12, and TNF-α were measured in the wild-type, NLRP3^-/-^, and caspase-1/11^-/-^ macrophages after *V. alginolyticus* infection. The results confirmed that the protein levels of IL-1β, IL-6, IL-12, and TNF-α remarkably reduced in the supernatant of NLRP3^-/-^ and caspase-1/11^-/-^ macrophages compared with the supernatant of WT macrophages infected with *V. alginolyticus* at 12 h (^***^ *p* < 0.001, **Figure 5D**). The validation of gene deletion efficiency in macrophages from mice that lacked NLRP3 and caspase-1/11 was performed in **Figures S4A, B**. Overall, these results illustrated that blocking the NLRP3 inflammasome downregulated *V. alginolyticus-*triggered inflammatory response in mouse macrophages.

## Discussion

*V. alginolyticus* is a zoonotic pathogen that settled in temperate, tropical, and subtropical marine ecosystems, with a frighteningly prolonged pathogenic period due to global warming. According to reports, *V. alginolyticus* causes inflammation in humans, which includes gastroenteritis, otitis media, otitis externa, and septicaemia in severe cases (Janda et al., 1986; Baker-Austin et al., 2018). However, the potential mechanism of inflammation induced by *V. alginolyticus* has not been reported. In this study, we established a model of *V. alginolyticus* infection of primary mouse peritoneal macrophages and used qPCR and other techniques to clarify the specific mechanism of *V. alginolyticus* activation of host innate immunity, providing a new target for clinical treatment. Nevertheless, there are still weaknesses in the present study compared with other *Vibrio* research. The role of other types of inflammasomes (enhanced in the transcription levels) in the inflammatory response of mouse macrophages induced by *V. alginolyticus* infection has not been thoroughly investigated.

When organisms are invaded by exogenous pathogens, the innate immune system is the first defense line to resist against injury. Innate immune cells, such as macrophages, exert their function through releasing cytokines and then recruiting more macrophages to aggregate, triggering inflammation. Inflammation occurs as a kind of host immune response to attempt to eliminate pathogens (Kuprash and Nedospasov, 2016). Excessive inflammation, however, can damage the organisms. The expression levels of inflammatory cytokines are closely related to the inflammatory responses. *V. parahaemolyticus* triggered the upregulation of IL-1β in mouse bone marrow macrophages (Higa et al., 2013). The levels of pro-inflammatory cytokines of IL-6 and TNF-α were significantly increased in *V. vulnificus-*infected RAW264.7 macrophages (Qin et al., 2019). *V. alginolyticus-*infected mice displayed inflammatory damage in liver and lung through HE detection, and cytokines of IL-1β and IL-6 were released in the serum *via* ELISA measurement (Liu et al., 2014). However, *V. alginolyticus* infection mechanisms were poorly understood. Therefore, the present study established a *V. alginolyticus* infection model *in vitro* using mouse peritoneal macrophages. The release of pro-inflammatory cytokines, including IL-1β, IL-6, IL-12, TNF-α, IL-17, IL-18, IFN-γ, and anti-inflammatory cytokine IL-10, was detected. Results showed that the transcription levels of various cytokines were significantly upregulated and cytokines of IL-1β and IL-6 occupied the highest levels; interestingly, the anti-inflammatory cytokine of IL-10 was also significantly secreted, which indicated that *V. alginolyticus* continuously stimulated macrophages during the monitored infection time. Moreover, an interesting phenomenon here is the production of IL-17, because IL-17 is released by Th17 cells. As we know, IL-17 secretion is regulated by IL-23. IL-23R is expressed on activated T cells, ILCs, γδ T cells, macrophages, and DCs (Kastelein et al., 2007; Awasthi et al., 2009; Aychek et al., 2015). The study of Hou et al. demonstrated that IL-23 induced mouse peritoneal macrophages to produce IL-17 under the stimulation of LPS (Hou et al., 2018). Overall, these data supported that the *V. alginolyticus in vitro* infection model was successfully established with mouse macrophages.

Pro-IL-1β was cleaved either by the classical caspase-1 pathway or by the non-classical caspase-11 pathway before they can be secreted outside the cell. It is worth noting that, in the current study, activation of caspase-1 is not the only one responsible for IL-1β secretion (Kayagaki et al., 2011). Western blotting results showed that IL-1β secretion dropped sharply but was not completely eliminated after blocking caspase-1 activation with Ac-YVAD-CHO. IL-1β released could not be detected only under the treatment of Z-VAD-FMK (a pan-caspase inhibitor). Our results suggest that there is a non-classical caspase-11-cleaving pathway in addition to the classical caspase-1-cleaving pathway for IL-1β. The ELISA results clarified that IL-1β release levels were still observed under Z-VAD-FMK treatment, implying that other inflammasomes are involved in IL-1β secretion. According to previous studies, *V. parahaemolyticus*-mediated inflammation is dominated by NLRP3 and NLRC4 inflammasomes. In this study, various PRRs were upregulated, including NOD1, NOD2, NLRP1, NLRP3, NLRC4, NLRC5, NLRC6, NLRP12, and AIM2. Among them, NLRP3 increased to the highest levels. Unfortunately, the role of other types of inflammasomes in the inflammatory response of mouse macrophages induced by *V. alginolyticus* infection has not been thoroughly investigated. Results of qPCR, Western blotting, and immunofluorescence revealed that the protein expression level of NLRP3 was significantly higher than that in untreated cells after *V. alginolyticus* infection; meanwhile, the location of NLRP3 displayed a puncta-like surrounding nucleus. Then, the characteristics of the NLRP3 inflammasome in IL-1β release were explored. Glyburide, a potassium channel inhibitor, could block the outflow of potassium ions and inhibit the assembly of the NLRP3 inflammasome (Zahid et al., 2019). Similarly, CA-074 methyl ester, an inhibitor of cathepsin B, restricted the release of the protease and blocked the activation of the NLRP3 inflammasome (Newman et al., 2009). In this study, macrophages either pretreated with glyburide or CA-074 Me resulted in a sharp decrease in IL-1β secretion; meanwhile, there was no significant difference in the protein expression levels of NLRP3. These results indicated that both potassium outflow and lysosomal rupture were involved in the activation of *V. alginolyticus*-mediated NLRP3 inflammasome activation. Moreover, to the best of our knowledge, hemolysin, as one of the most important virulence factors of *vibrios*, plays indispensable roles in the activation of NLRP3 inflammasome in *Vibrio*-infected mouse macrophages (Higa et al., 2013; Song et al., 2015). We hypothesized that the hemolysin of *Vibrio alginolyticus* significantly contributed to the activation of NLRP3 in mouse peritoneal macrophages. And that will be the focus of our next research.

It has recently been reported that the secretion of IL-1β induced by gram-negative pathogens such as *Shigella* (Li et al., 2020) and *Pseudomonas aeruginosa* (Deng et al., 2015) when they infected macrophages is related to infection doses and time. Therefore, the relationship between the releases of IL-1β after the stimulation of mouse macrophages by *V. alginolyticus* was determined by monitoring different MOIs and time. Our results showed that IL-1β secretion peaked when MOI was 1. The amount of IL-1β decreased when the MOI increased to 10. However, in the *Shigella* and *Pseudomonas aeruginosa* studies, IL-1β rose linearly along with increasing infection doses. This may probably be due to excessively high concentrations of *V. alginolyticus* which could cause mouse macrophage death in a short period of time (**Figure S5**), as well as degradation of IL-1β secretion over time. In the time-dependent experiment, the performance of *V. alginolyticus* is consistent with that of other Gram-negative bacteria, and the production of IL-1β increased with the extension of stimulation time. Interestingly, the transcription level of IL-1β decreased with time, which was consistent with the mRNA transcription levels both in lipopolysaccharide (LPS)-stimulated bone marrow-derived macrophages and in RAW264.7 macrophages, reported by Dagmar Schilling (Schilling et al., 2001). Overall, the secretion of IL-1β mediated by the NLRP3 inflammasome was partly in a dose- and time-dependent manner.

**Figure 6:**
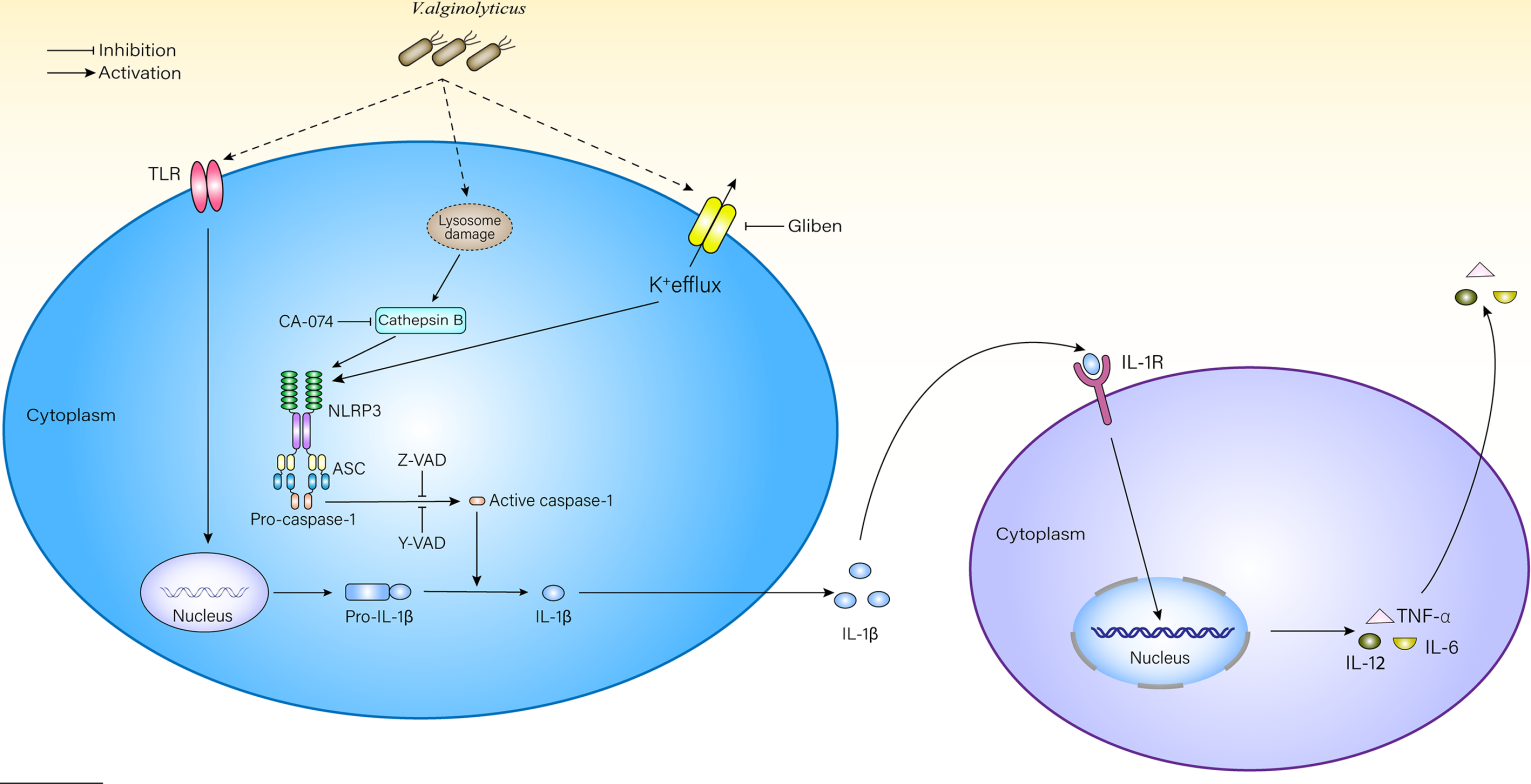
Schematic diagram of mechanisms underlying *V. alginolyticus*-induced inflammatory response. *V. alginolyticus* triggering inflammatory response by recognizing extracellular TLRs and intracellular NLRP3. NLRP3 inflammasome was activated by potassium ion outflow and lysosomal disruption to release cathepsin B, mediated IL-1β release, and controlled cytokine secretion. This process could be completely reversed by Z-VAD-FMK or partially reversed by Ac-YVAD-CHO, glyburide, and CA-074 methyl ester.

This excess IL-1β outside cells may recruit more macrophages to participate in the innate immune response *via* the IL-1β receptor (IL-1R) on the surface of macrophages (Dinarello, 2018). After receiving signals from IL-1R, macrophages would feed it back to the nucleus and activate the NF-κB or MAPK signaling pathways, promote the transcription of pro-inflammatory factors and chemokines, and further aggravate the inflammatory response (Boraschi et al., 2018) (**Figure 6**). The model of *V. alginolyticus-*infected mouse peritoneal macrophages was established *in vitro*, inhibitors assays showed that the production of IL-1β is inhibited, and the processing of IL-6, IL-12, and TNF-α was also affected in this study. The transcription level of IL-12 did not obviously change at 2–6 h. Therefore, we detected IL-12 mRNA levels at 12 h post infection and found that the level of IL-12 was increased compared with the control group (**Figure S6**). To further validate our results, NLPR3^-/-^ and caspase-1/11^-/-^ mouse macrophages were used in this experiment. The results showed that IL-1β levels were obviously decreased compared with WT, which may lead to decreased IL-1β and IL-1R binding and further affect the production pathways of inflammatory cytokines, resulting in significantly decreased IL-6, IL-12, and TNF-α release levels. Therefore, blocking the NLRP3 inflammasome in macrophages could remit *V. alginolyticus*-mediated inflammatory response.

Collectively, the present study firstly demonstrated that *V. alginolyticus* induced inflammatory response in mouse macrophages through outflow of potassium ions and lysosomal rupture-triggered NLRP3 inflammasome signal pathway activation; NLRP3 inflammasome-mediated caspase-1 activation was closely related to IL-1β p17 release; and blocking the NLRP3 inflammasome attenuated downstream pro-inflammatory cytokine secretion in response to *V. alginolyticus* infection. This study may provide new targets for the development of potential anti-inflammatory treatment strategies against *V. alginolyticus* infection.

## Data Availability Statement

The original contributions presented in the study are included in the article/**Supplementary Material**. Further inquiries can be directed to the corresponding authors.

## Ethics Statement

The animal study was reviewed and approved by the Animal Welfare and Research Ethics Committee, Jiangsu Ocean University, certificate number 2019220341.

## Author Contributions

JD designed the research. JW, QD, QY, and GY conducted the research. JW, QD, HF, FL, QY, XZ, and TZ analysed the data. JW, QD, PZ, FL, and GL wrote the manuscript. BB edited the English language. JD, GL, and PZ directed the project. All authors contributed to the article and approved the submitted version.

## Funding

The authors thank the Open Foundation of Jiangsu Key Laboratory of Marine Bioresources and Environment (JSIMR202016), the Jiangsu Distinguished Professor program (KK19515), the Postgraduate Research & Practice Innovation Program of Jiangsu Ocean University, and the Priority Academic Program Development of Jiangsu Higher Education Institutions of China for financial support. They had no role in the study design, data collection and analysis, decision to publish, or preparation of the manuscript.

## Conflict of Interest

The authors declare that the research was conducted in the absence of any commercial or financial relationships that could be construed as a potential conflict of interest.

## Publisher’s Note

All claims expressed in this article are solely those of the authors and do not necessarily represent those of their affiliated organizations, or those of the publisher, the editors and the reviewers. Any product that may be evaluated in this article, or claim that may be made by its manufacturer, is not guaranteed or endorsed by the publisher.
