# Supplementary material for: Vibrio alginolyticus Triggers Inflammatory Response in Mouse Peritoneal Macrophages via Activation of NLRP3 Inflammasome
Source: Front Cell Infect Microbiol. 2021 Nov 15;11:769777. doi: 10.3389/fcimb.2021.769777 (PMC8634873; doi:10.3389/fcimb.2021.769777)
Supplement: Supplementary file 1 [file DataSheet_1.pdf]

FIGURE S1

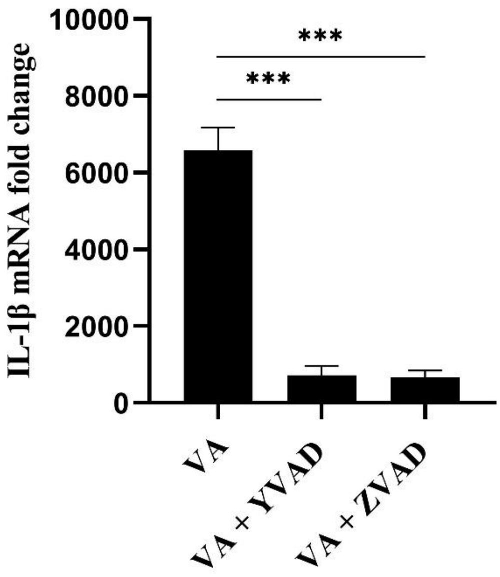

FIGURE S1 | Macrophages were pretreated with 100 μM Ac-YVAD-CHO or 10 μM Z-VAD-FMK for 1 h, and then infected with *V. alginolyticus* at MOI of 1 for 12h, the mRNA transcription level of IL-1β/p17 was detected by qPCR. Data were representative of at least three repetitive experiments. ***$p < 0.001$.

FIGURE S2

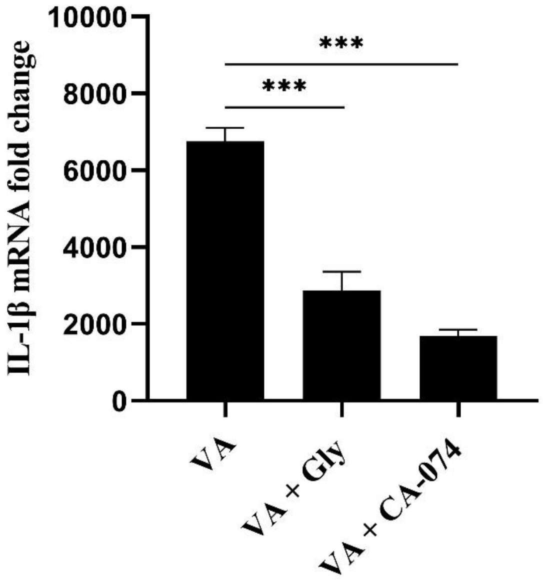

FIGURE S2 | Mouse peritoneal macrophages were pre-treated with 50 μM Glyburide or 25 μM CA-074 methyl ester for 1 h, and then infected with *V. alginolyticus* at MOI of 1 for 12 h. The mRNA transcription level of IL-1β was examined by qPCR. Data were representative of at least three repetitive experiments. ***$p < 0.001$.

FIGURE S3

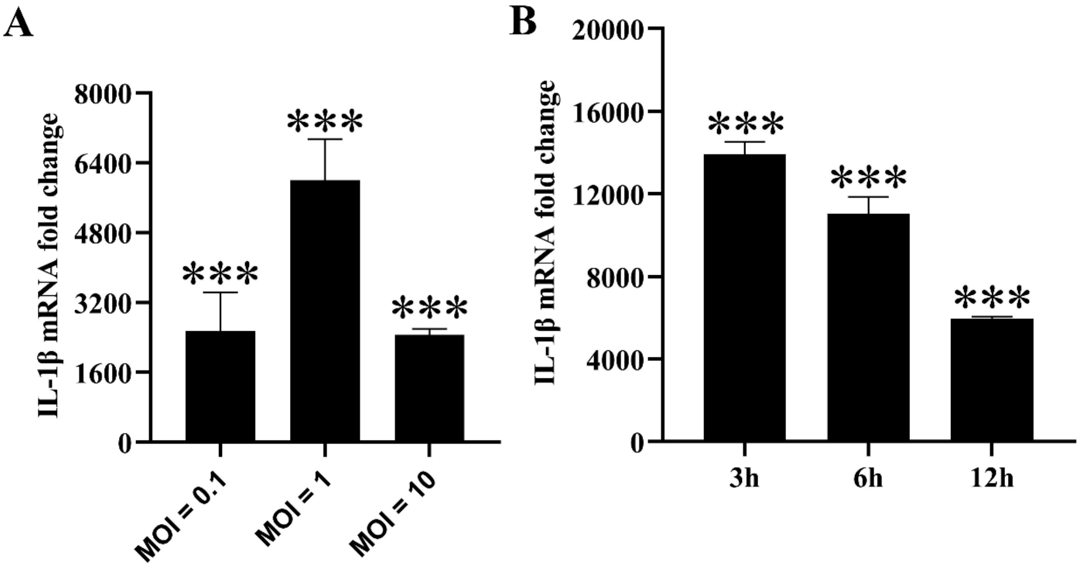

FIGURE S3 | (A) Dose-dependent (MOI = 0.1, 1, and 10) assay of macrophages infected with *V. alginolyticus* for 12 h. The mRNA transcription level of IL-1β was confirmed by qPCR. (B) Time course (3, 6, and 12 h) assay of macrophages infected with *V. alginolyticus* at MOI of 1. The mRNA transcription level of IL-1β was confirmed by qPCR. Data were representative of at least three repetitive experiments. ***$p < 0.001$.

FIGURE S4

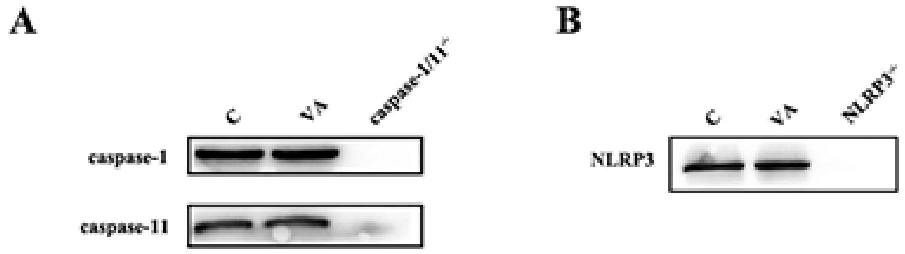

FIGURE S4 | (A) The caspase-1 and caspase-11 protein expression levels were detected using western blotting in cell lysates from uninfected wild type, VA-infected wild type and caspase-1/11$^{-/-}$ mouse macrophages. (B) The NLRP3 protein expression levels were detected using western blotting in cell lysates from uninfected wild type, VA-infected wild type and NLRP3$^{-/-}$ mouse macrophages.

FIGURE S5

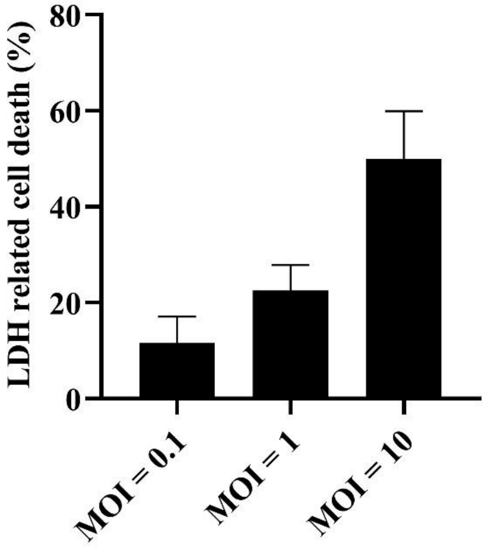

FIGURE S5 | Cell death was monitored by measuring LDH release in supernatants after infection with *V. alginolyticu* (MOI= 0.1,1 or 10) for 12 h. Data were representative of at least three repetitive experiments.

FIGURE S6

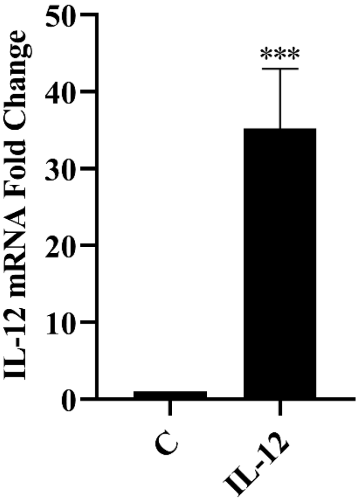

FIGURE S6 | Macrophages were infected with *V. alginolyticus* at an MOI of 1 for 12 h, and then mRNA levels of various inflammatory cytokines were tested using real-time PCR.
